# Audit of electronic operative documentation in interventional radiology: the value of standardised proformas

**DOI:** 10.1186/s42155-020-00163-w

**Published:** 2020-09-23

**Authors:** Iakovos Theodoulou, Rhys Judd, U. Raja, N. Karunanithy, Tarun Sabharwal, Afshin Gangi, Athanasios Diamantopoulos

**Affiliations:** 1 Department of Interventional Radiology, Guy’s and St. Thomas’ NHS Foundation Trust, St Thomas’ Hospital, 1st floor, Lambeth Wing, Westminster Bridge Road, London, SE1 7EH UK; 2 North Shore Hospital, Waitemata DHB, Auckland, New Zealand; 3 School of Biomedical Engineering & Imaging Sciences, Faculty of Life Sciences & Medicine, Kings College London, London, UK; 4 Department of Interventional Radiology, Nouvel Hôpital Civil, Hôpitaux Universitaires de Strasbourg, 1, place de l’ Hôpital, 67000 Strasbourg, France

## Abstract

**Background:**

On the background of the interventional radiology department of a tertiary hospital converting its periprocedural documentation from paper-based to electronic using a standardised proforma, a study was performed to ascertain the effects of this change on the standard of clinical documentation for radiologically-guided angiographic procedures. Using a retrospective approach, perioperative records were analysed in reverse chronological order for inclusion in the study. The standard for this audit was developed in the form of minimum criteria that all clinical documentation of angiographic procedures were expected to meet.

**Results:**

The audit was performed at three equally spaced intervals of 6 months, yielding a total of 99 records. The baseline audit of paper-based records concluded > 80% completeness for 8 out of the 14 of parameters measured, with only two of parameters meeting the target of 100% completeness. The second audit cycle performed on electronic records found 7 out of 14 parameters demonstrating absolute improvement in completeness, when compared to paper-based, but with the number of parameters exceeding 80% completeness falling to only 4 out of 14. Again, 100% completeness was observed in only 2 of the parameters. In the final audit cycle, after the introduction of a standardised electronic proforma, performance improved in every dimension with 6 out of 14 parameters reaching completeness of 100% and the 80% completeness threshold met by 12 out of 14 parameters.

**Conclusion:**

The construction of a procedure-specific perioperative electronic proforma can save clinicians valuable time and encourage safe and effective clinical documentation.

## Background

Effective communication is of paramount importance in healthcare service provision, with accurate clinical documentation forming the foundation of safe, transparent and auditable clinical practice. The operation note plays a crucial role in enabling clear communication and continuity of care between the operating team and the rest of the team involved in the care of patients. It is essential to providing safe and comprehensive care from admission to discharge. It is therefore crucial that clinical records are clearly organised and contain all clinically relevant information (McManus et al. 2000). This is becoming more pertinent in an ever increasing litigious medical landscape (Omary et al. 2003).

In recent times, interventional radiology (IR) has seen a significant expansion in its scope and complexity of practice. Resultantly, this has led to a gradual shift in responsibility from service provision for other clinical specialities to IR taking ownership of the peri and post-operative care for patients treated. This paradigm shift is reinforced by the British Society of Interventional Radiology’s (BSIR) recent vote for IR to be granted its own specialty status in the United Kingdom – even after only recently having been granted subspecialty status (Theodoulou et al. 2020). Coupled with the rapidly increasing number of image-guided interventions available, it follows that appropriate documentation of periprocedural care is fundamental both for successful patient-centred care and support for the autonomy of IR as a specialty (Kohi et al. 2015). This is also in line with requirements as set out by the Joint Commission on the Accreditation of Healthcare Organizations (Novitsky et al. 2005).

On the background of the IR Department of a tertiary hospital converting its periprocedural documentation from paper-based to electronic, an internal pilot study was performed to ascertain the effects of this change on the standard of clinical documentation for radiologically-guided angiographic procedures. Data collected from the first audit cycle provided the basis for the implementation of an electronic proforma that aimed to streamline periprocedural documentation, ensuring that all relevant information was included in each clinical report.

This study proposes two main aims. Firstly, to highlight and reinforce previously supported notions about the role of electronic patient records in modern healthcare. Secondly, to assess the value of electronic proformas in the accuracy and completeness of periprocedural documentation in patients undergoing either diagnostic or therapeutic angiographic procedures in IR departments.

## Methods

### Intervention

Changes implemented consisted of transition from paper based to electronic records and secondly the implementation of an electronic proforma. The latter was created in the form of a text-based template, that enabled a more guided approach into documenting perioperatively. The operator would copy and paste this template into the field normally filled in with a free-text report.

### Inclusion of records

Using a retrospective approach, perioperative records were analysed in order of recency (most recent to oldest) for inclusion in the study using the following criteria:

- Completion of a radiologically-guided angiographic procedure within the study timeframe at St Thomas’ Hospital in London.
- Availability of adequate documentation, which required a minimum of a post-operative note completed by one of the operating interventional radiologists.

Multiple angiographic procedures performed on the same patient were counted as different instances of clinical documentation and were recorded as such. If a case met the inclusion criteria, the entire perioperative record was retrieved and examined against the minimum criteria as set out by *The Standard* below (Table 1). Although there was a focus on proceduralist completion of records, information was still marked as available if this was documented elsewhere in the patient’s records. For example, completion of the WHO checklist was marked as complete even if this was documented by nursing staff, as opposed to the operating interventional radiologist. Overall, the audit was performed in three stages of equally spaced intervals of 6 months. Each audit cycle aimed to yield 25 records, resulting in a total of 99 records being included in the study. Between the second and third audit cycles, an effort was made to increase engagement of clinicians with reporting and maximise the filling in of all necessary fields.

**Table 1:** Standard of assessment for perioperative documentation

The Standard	
**Pre-operative**	●Name and signature of operator(s); ●Documentation of type of consent obtained (verbal vs written); ●Record of WHO safety checklist completed.
**Intra-operative**	●Name and quantity of medications used; ●Site of puncture recorded; ●Presence/Absence of complications recorded; ●Management of complications recorded (if any).
**Post-operative**	●Plan for post-operative vital monitoring recorded; ●Required duration of bed rest recorded; ●Puncture site instructions recorded; ●Distal pulse monitoring instructions recorded; ●Requirements for anti-coagulation recorded; ●Requirements for post-operative oral intake recorded; ●Follow-up plans recorded.

### Assessment of records

The *standard* for this audit was developed in the form of a set of minimum criteria that all clinical documentation of radiologically guided angiographic procedures should meet. These were established following discussion amongst the authors and after consulting both the Royal College of Surgeons Good Surgical Practice Guidelines (England RCoSo 2002) and the WHO Guidelines for Safe Surgery (Lives 2009). The relevant criteria were then modified to specifically align with radiologically-guided angiographic procedures performed at the current tertiary centre. Resultantly, a set of minimum criteria was established as illustrated in Table 1. It should be noted that in order to meet the *‘Name & Signature’* criterion for an electronic record, only the operators’ names where required due to the inability to electronically sign documents. Legibility was assumed to be adequate for all electronic records as these were all typed and thus easily discerned. Assessment of records was performed twice by two of the authors, and any discrepancies were resolved by one of the senior authors.

The conduction of two audit cycles reflects the implementation of two subsequent interventions, that is, the conversion to electronic records followed by a second change; the implementation of a specific proforma within electronic records. By performing these audit cycles, the authors aimed to isolate the effects of each intervention such that appropriate effect sizes, if any, would be attributed to the relevant intervention.

### Statistical analysis

Descriptive statistics were used to highlight overall trends and non-parametric statistical tests were applied to explore further associations between categorical variables using Chi-squared and/or Fisher exact tests, where appropriate. *P*-values were set at 0.05 in order to achieve statistical significance. All statistical analyses were performed using SPSS (SPSS Statistics for Mac, Version 16.0. Armonk, NY: IBM Corp).

## Results

**Fig. 1:**
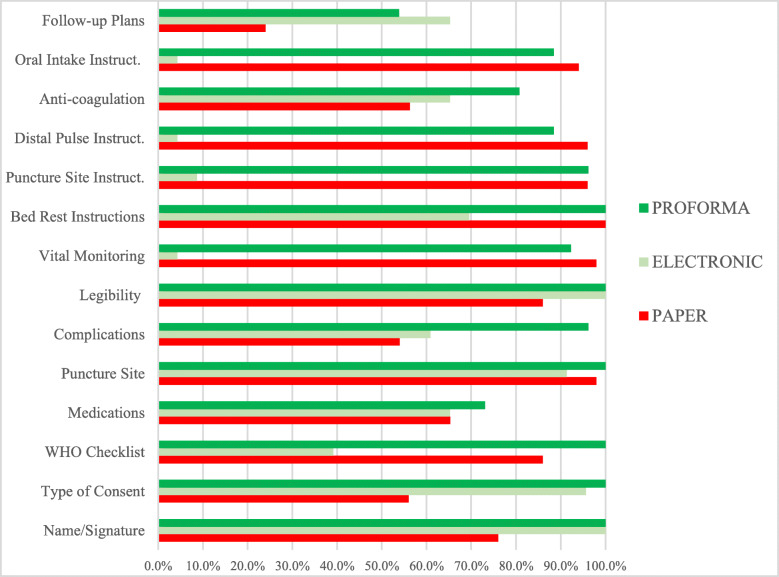
Distribution of perioperative completeness of records across 14 parameters

A total of 99 records were included in the study, all of which were assessed against the agreed standard (Table 2, Fig. 1). The study duration was 18 months, during which, perioperative documentation at the IR department underwent a number of changes, namely conversion to electronic patient records followed by implementation of an electronic proforma.

**Table 2:** Assessment of perioperative records over a period of 18 months

Standard:	1st Audit	2nd Audit			3rd Audit				
	Paper records	Electronic records			Electronic records with proforma				
		Electronic	Vs ***Paper***		Proforma	Vs ***Paper***		Vs ***Electronic***	
	*n (%)*	*n (%)*	*Absolute change (%)*	*p-value*	*n (%)*	*Absolute change (%)*	*p-value*	*Absolute change(%)*	*p-value*
Name & signature noted	38 (76)	23 (100)	+ 24	0.008	26 (100)	+ 24	0.006	–	–
Type of consent recorded	28 (56)	22 (96)	+ 40	< 0.001	26 (100)	+ 44	< 0.001	+ 4	0.469
WHO checklist Completed	43 (86)	9 (39)	−47	< 0.001	26 (100)	+ 14	0.088	+ 17	< 0.001
Medications administered	32 (64)	15 (65)	+ 1	1.000	19 (73)	+ 9	0.606	+ 8	0.757
Puncture site noted	49 (98)	21 (91)	−7	0.232	26 (100)	+ 2	1.000	+ 9	0.215
Complications recorded	27 (54)	14 (61)	+ 7	0.621	25 (96)	+ 42	< 0.001	+ 35	0.003
Legibility of records	43 (86)	23 (100)	+ 14	0.090	26 (100)	+ 14	0.088	–	–
Vital monitoring recorded	49 (98)	1 (4)	− 94	< 0.001	24 (92)	−6	0.268	+ 88	< 0.001
Bed rest instructions	50 (100)	16 (70)	− 30	< 0.001	26 (100)	–	–	+ 30	0.003
Puncture site instructions	48 (96)	2 (9)	−93	< 0.001	25 (96)	–	1.000	+ 87	< 0.001
Distal pulse instructions	48 (96)	1 (4)	−92	< 0.001	23 (88)	−8	0.331	+ 84	< 0.001
Anti-coagulation regime	27 (54)	15 (65)	+ 11	0.449	21 (81)	+ 27	0.026	+ 16	0.332
Oral Intake instructions	47 (94)	1 (4)	−90	< 0.001	23 (88)	−6	0.406	+ 84	< 0.001
Follow-up plans	12 (24)	15 (65)	+ 41	0.001	14 (54)	+ 30	0.012	−11	0.562
**Total**	**50 (100)**	**23 (100)**			**26 (100)**				

Note: (−) is due to missing p-values as there was no difference across groups

### First audit: paper-based records

The first audit, performed on paper-based records, revealed encouraging performance for more than half of the parameters of the standard – scoring more than 80% completeness in 8 out of 14 of parameters. While the aim was to reach 100% completeness in all parameters this was not met in all but two parameters: ‘*Name & Signature’* and *‘Bed rest instructions’*. Certain parameters performed better than others; for example, the parameters that comprised post-operative care instructions exceeded, on average, 90%. Stemming from this first assessment, important omissions were highlighted and formed an important target for subsequent audits. For example, the lowest scores were observed in *‘Type of consent’*, *‘Complications’* and *‘Anticoagulation regime’* with scores of 56%, 56% and 54%, respectively. Attempts to attribute instances of omissions to specific time periods, such as the pre-operative or post-operative documentation, did not reveal any significant associations.

### Second audit: electronic records

Following the transition from paper-based to electronic records, the standard was revisited to assess for improvements in the documentation parameters. Whilst 7 out of 14 parameters demonstrated improvement in completeness of records, the total number of parameters exceeding 80% completeness fell to 4 out of 14. Similar to the first audit cycle, 100% completeness was observed in only 2 parameters. Poorest performance was noted in the post-operative records with 4 parameters demonstrating completeness of less than 10%. Statistically significant improvements were observed across a number of parameters such as *‘Name & Signature’* and *‘Type of consent’* and *‘Follow-up plans’* with *p*-values of 0.008, < 0.001 and 0.001, respectively. Significant decreases were also observed.

### Third audit: electronic proforma records

The final audit sought to identify changes in the parameters 12 months post-conversion to electronic records and 6 months following implementation of the electronic proforma with predefined fields. Performance improved in every dimension when compared to the previous 2 audit cycles. Completeness of 100% was observed in 6 out of 14 parameters, while the 80% completeness threshold was met for all but 2 parameters (‘*Medications’* and *‘Follow up plans’*). Comparisons against each of the previous audit cycles revealed improvements which, in some instances, also reached statistical significance (Table 2).

## Discussion

IR operations are rapidly becoming an essential part of clinical management strategies for a variety of conditions; ranging from embolization approaches in the management of lower gastrointestinal bleeds (Oakland et al. 2019) or post-partum haemorrhage (Lindquist and Vogelzang 2018) to endovascular approaches for abdominal aortic aneurysm repairs (Chaikof et al. 2009). With the emergence and widespread endorsement of these techniques, there comes the relevant clinical responsibility. The extent to which this responsibility falls onto radiologists remains largely ambiguous and is often discordant between departments, hospitals and geographical areas. Nevertheless, the abundance of radiologically-guided procedures occurring daily in hospitals mandates a comprehensive approach to patient care, from the moment of vetting a referral to the point of discharging the patient from the IR department. Clinical documentation is a vital part, capturing the patient’s journey. In addition, it provides a measure of the performance of IR as a specialty and is an important part of maintaining a consistently high standard of care.

The aim of this pilot study was to establish the baseline performance of the current department with regards to clinical documentation and highlight, where appropriate, potential areas for improvement to maximise patient safety and continuity of care. In doing so, this provided a platform upon which an electronic proforma was created containing appropriate prompts to facilitate consistent, concise and accurate documentation for the completing clinician. The effects of which are implicit in the marked improvement of perioperative documentation completeness over the period of 18 months. Whilst there was a noticeable drop in performance following transition to electronic records, with several parameters suffering significantly, we attribute this to transitional adjustments where operators were only beginning to appreciate the way electronic records were being utilised as part of their new operative routine. This speculation is in fact reinforced by the results of the third audit, 18 months later, where improvements were observed across almost all parameters, suggesting that electronic records are indeed helpful and even more so in the presence of a proforma. On extrapolation to auditing processes in general, it is advisable to allow for a longer transition period for operators to familiarise themselves with the new record keeping system before re-assessment.

The stratification of study criteria into pre-, intra- and post-procedural information provided insight into whether there are specific timepoints in the patient journey where information collection and reporting may be lacking and thus requiring greater need for intervention. With the exception of post-operative documentation of the second audit, the other two audit cycles (paper-based and proforma) did not yield similar results. Although electronic transition, through the use of proforma, led to improvement in many of the documentation parameters, this does not entirely supersede the need for concurrent paper records for some of the parameters. For example, even though puncture site documentation was consistently excellent for both the paper-based and electronic records, the diagram on the front of the operative booklet for drawing the puncture site appeared to work well for most clinicians and aided immediate post-operative care. Furthermore, the consistently lower documentation rate for *‘Medications’* across all audit cycles, reflects more the fact that information remains dispersed rather than absent. A medication chart was present and completed in all patients’ operative booklets – however, identifying this was often problematic and time-consuming. The medication chart section in the proforma aims to encourage clear and more consistent medication documentation, with the downside, however, of often requiring duplicating clinical data. Complication recording was relatively low for both the paper-based and electronic records – a figure which improved dramatically with the proforma. In cases in which it was not appropriately documented, it was assumed that this was largely due to mere absence of complications.

Proformas have traditionally played an important role in streamlining clinical documentation and smoothing out inconsistencies across departments. For example, as Barritt et al. recently showed, the use of procedure-specific computerised proformas for hemi-arthroplasty operations significantly improved the quality of reports to meet The Royal College of Surgeons of England guidelines (Barritt et al. 2010). Similarly, Laflamme et al. concluded that electronic note templates are superior to dictation services in improving both the efficiency and the comprehensiveness of perioperative documentation (Laflamme et al. 2005). A further benefit of proforma use includes the reduction in reporting variability such that greater transparency is achieved when it comes to reimbursement of procedures (Taslakian and Sridhar 2017).

Whilst this pilot has yielded encouraging results, it must be remembered that the scope of this audit was limited to radiologically-guided angiographic procedures. This limits the generalisability of the specific template to other IR procedures. Moreover, given that this is only a draft template, it will likely undergo further long-term evaluation before its official implementation. The study would benefit from longer-term data including bigger samples and use across different hospital sites. It would be interesting to observe performance across district general hospitals where IR departments are smaller, with a varied daily workload and with potentially fewer angiographic procedures per day.

## Conclusion

A thoughtfully constructed electronic proforma for specific procedures can save clinicians valuable time when completing perioperative documentation and ensure that all relevant clinical information is recorded. The advantages intrinsically linked to the use of electronic records sometimes cannot be conferred fully unless appropriate adjustments are introduced to account for the needs of the relevant specialty. Angiographic IR procedures are one such example in which the presence of an electronic proforma appears to produce more consistent perioperative clinical documentation.

## Data Availability

The datasets used and/or analysed during the current study are available from the corresponding author on reasonable request.
